# DIRMC: a database of immunotherapy-related molecular characteristics

**DOI:** 10.1093/database/baae032

**Published:** 2024-05-06

**Authors:** Yue Liu, Yuhuan Zhou, Xiumei Hu, Wuri Le-Ge, Haoyan Wang, Tao Jiang, Junyi Li, Yang Hu, Yadong Wang

**Affiliations:** Faculty of Computing, Harbin Institute of Technology, Harbin, Heilongjiang 150001, China; School of Computer Science and Technology, Harbin Institute of Technology (Shenzhen), Shenzhen, Guangdong 518055, China; Beidahuang Industry Group General Hospital, Harbin 150001, China; Department of Pain, Tongliao City Hospital, Tongliao 028000, China; Faculty of Computing, Harbin Institute of Technology, Harbin, Heilongjiang 150001, China; Faculty of Computing, Harbin Institute of Technology, Harbin, Heilongjiang 150001, China; School of Computer Science and Technology, Harbin Institute of Technology (Shenzhen), Shenzhen, Guangdong 518055, China; Faculty of Computing, Harbin Institute of Technology, Harbin, Heilongjiang 150001, China; Faculty of Computing, Harbin Institute of Technology, Harbin, Heilongjiang 150001, China

## Abstract

Cancer immunotherapy has brought about a revolutionary breakthrough in the field of cancer treatment. Immunotherapy has changed the treatment landscape for a variety of solid and hematologic malignancies. To assist researchers in efficiently uncovering valuable information related to cancer immunotherapy, we have presented a manually curated comprehensive database called DIRMC, which focuses on molecular features involved in cancer immunotherapy. All the content was collected manually from published literature, authoritative clinical trial data submitted by clinicians, some databases for drug target prediction such as DrugBank, and some experimentally confirmed high-throughput data sets for the characterization of immune-related molecular interactions in cancer, such as a curated database of T-cell receptor sequences with known antigen specificity (VDJdb), a pathology-associated TCR database (McPAS-TCR) *et al*. By constructing a fully connected functional network, ranging from cancer-related gene mutations to target genes to translated target proteins to protein regions or sites that may specifically affect protein function, we aim to comprehensively characterize molecular features related to cancer immunotherapy. We have developed the scoring criteria to assess the reliability of each MHC–peptide–T-cell receptor (TCR) interaction item to provide a reference for users. The database provides a user-friendly interface to browse and retrieve data by genes, target proteins, diseases and more. DIRMC also provides a download and submission page for researchers to access data of interest for further investigation or submit new interactions related to cancer immunotherapy targets. Furthermore, DIRMC provides a graphical interface to help users predict the binding affinity between their own peptide of interest and MHC or TCR. This database will provide researchers with a one-stop resource to understand cancer immunotherapy-related targets as well as data on MHC–peptide–TCR interactions. It aims to offer reliable molecular characteristics support for both the analysis of the current status of cancer immunotherapy and the development of new immunotherapy. DIRMC is available at http://www.dirmc.tech/.

**Database URL**: http://www.dirmc.tech/

## Introduction

Cancer is a global health challenge that knows no boundary (1). Over the next 20 years, the number of new cancer cases worldwide is expected to increase by about 50% (2). Despite the rapid advancement in medical technology, the mortality rate of patients and the societal burden remain high (3). Traditional methods such as surgery, radiotherapy (4), targeted therapy and chemotherapy (5) can effectively inhibit some tumors, but there are still many cancer patients who cannot benefit from them. Currently, cancer immunotherapy (6), which uses immune pathways to treat cancer, is rapidly becoming a recognized pillar of cancer treatment alongside surgery, chemotherapy, radiotherapy and targeted therapy (7). Cancer immunotherapy has been shown to be remarkably successful by eliciting a systemic anti-tumor T-cell response in the host (8). Forms of cancer immunotherapy, including oncolytic virus therapies, cancer vaccines, cytokine therapy, adoptive cell therapies (ACTs), and immune checkpoint inhibitors (ICIs), have been developed and shown promise in clinical practice. Among them, ACTs and ICIs have achieved durable clinical responses (6, 9). However, the overall disease remission remains low and only effective for a subset of cancer patients (10). Therefore, identifying more viable candidate targets is essential to provide more patients with the opportunity to benefit from cancer immunotherapy.

Currently, with the rapid development of research in this field, a large amount of data related to cancer immunotherapy has been accumulated in a short period of time. This includes the development of prediction tools for peptide of the target protein–T-cell receptor (TCR) interactions, such as peptide-TCR matching prediction (ERGO) (11), sequence-based prediction of peptide-TCR binding (NetTCR-2.0) (12), as well as the establishment of databases related to cancer immunotherapy, such as a curated database of T-cell receptor sequences with known antigen specificity (VDJDBdb) (13), the T cell receptor structural repertoire database (TCR3d) (14), database of structure-inferred antigenic epitopes (Epitome) (15) and the Immune Epitope Database (IEDB) (16). However, each of these databases has its own focus. For example, ERGO and NetTCR-2.0 provide webservers for TCR–peptide binding prediction. VDJdb is a database that stores and aggregates the results of published T-cell specificity assays and provides a universal platform that couples antigen specificities with TCR sequences. TCR3d is a resource containing all known TCR structures, with a particular focus on antigen recognition. Epitome is a database of all known antigenic residues and the antibodies that interact with them. IEDB includes immune epitope data related to all species studied and includes antibody, T cell and MHC binding contexts associated with infectious, allergic, autoimmune and transplant-related diseases, without cancer. These databases are crucial for the development of cancer immunotherapy. However, to our knowledge, no other dedicated resource has been devoted to collect and comprehensively characterize all the molecular characteristics associated with different types of cancer immunotherapy.

To fill this gap, we have characterized a comprehensive manually curated database called DIRMC, which contains 397 cancer-related genes, involving 4345 cancer-related mutations, 109 approved cancer immunotherapy against the cancer-related genes above and 1987 experimental data entries registered in ClinicalTrials.gov/DrugBank. All immunotherapy targets, along with 2706 immune-related peptides and TCR interactions, were manually curated by reading 5565 literatures and integrating high-throughput sequencing data resources. In addition, we provide predictive interfaces that allow users to predict the binding affinity of the peptide of interest to the MHC and TCR. We hope that DIRMC can serve as a valuable data resource for researchers to expand the indications of current cancer immunotherapy, discover additional candidate targets for cancer immunotherapy, and develop new immunotherapy that can benefit more patients. All the information in DIRMC is freely available at http://www.dirmc.tech/.

## Materials and methods

### Data collection

Our data sources are mainly in two directions, namely literature mining and publicly available data resources. With cancer immunotherapy as the keyword, 18 566 literature reviews on cancer immunotherapy in the past 5 years were screened in PubMed database. Through batch traversing their abstracts with keywords related to immunotherapy, we found that cancer immunotherapy can be categorized into six major classes, including non-specific immunotherapy, which contains cytokine therapies, ICIs, cancer vaccines, ACTs, targeted antibodies and oncolytic virus therapies (6, 17, 18). Each type of immunotherapy is centered around target proteins (including neoantigens) or peptides. By integrating data from multiple national drug approval platforms, including the U.S. Food and Drug Administration (FDA), National Medical Products Administration (NMPA), European Medicines Agency, HealthCanada, etc., and ClinicalTrials.gov, a resource for drugs or treatment regimens in clinical trials, as well as drug–target interaction databases like DrugBank (19) and the Drug-Gene Interaction database (DGIdb) (20), we have achieved a step-by-step expansion and integration of all information related to target proteins. We have obtained a comprehensive information flow of molecular characteristics related to immunotherapy, from mutation to gene to target protein to disease and ultimately to treatment strategies. The five core aspects of information involved are target proteins, genes translating the target proteins, potential pathogenic variants occurring on the genes, diseases associated with the target proteins, and corresponding treatment strategies.

**Figure 1.**
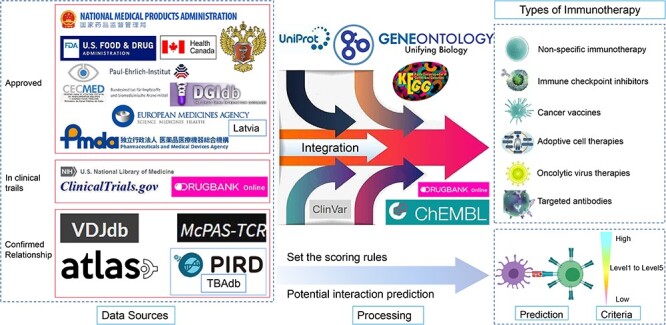
Flowchart of the DIRMC database. From left to right, there are three sections: data sources, data processing and the final database structure, including six types of immunotherapy developed, MHC–peptide and peptide–TCR binding affinity prediction and the reliability scoring system established. The data sources include approved immunotherapy for the target protein, immunotherapy targeting specific proteins that are still in the clinical trial phase and data resources related to these target proteins from experimentally confirmed or high-throughput sequencing data.

By integrating information from UniProt (21), Gene Ontology (GO) (22, 23), Kyoto Encyclopedia of Genes and Genomes (KEGG) (24), ClinVar (25), an open access bioactivity database (ChEMBL) (26), DrugBank and National Center for Biotechnology Information (NCBI) GenBank (27) and NCBI Gene (https://www.ncbi.nlm.nih.gov/gene) databases, we expanded the molecular features belonged to each aspect. For ACT therapy, in order to find out more reliable interactions between tumor-associated antigen peptides and their MHCs and TCRs, we explored three key sources: literature, ClinicalTrials.gov and validated database. The literature search included not only the review articles above but also 5565 articles identified by searching with keywords ‘CAR-T’ (chimeric antigen receptor-T cell therapy) and ‘TCR-T’ (engineered T cell receptor-T cell therapy). ClinicalTrials.gov was used to find relevant information on MHC–peptide–TCR interactions in TCR-T therapy and therapy about CAR-T within all clinical trials related to ACT therapy, as well as all registries related to targets of the other five immunotherapies. The main sources of the database include VDJdb, a manually curated database of TCRs and BCRs targeting known antigens (TBAdb),^1^ McPAS-TCR, and Altered TCR Ligand Affinities and Structures (ATLAS) datasets, which are primarily used to describe the sequences of immune receptors (TCRs and B-cell receptors (BCRs)) and their interactions with antigens reported in the literature or experimentally verified. In addition, we provide two personalized prediction interfaces that allow users to predict the binding affinity of peptides of interest to MHC or TCR. Users can select different data types to enter the corresponding IEDB analysis resources to predict the binding affinity between MHC I and peptide and MHC II and peptide. Users can also select different prediction methods, such as NetTCR (12), TCRMatch (28) and TCRex (29) to predict the binding affinity of TCR to the peptide of interest. We hope that this can further help screen potential immunotherapeutic target interactions. An overview of the database integration process is shown in Figure 1.

### Reliability criteria

To address the complexity of target interactions in ACTs, help researchers clearly understand the importance of the various types of target interactions and facilitate researchers to screen candidate priority sets of research targets, this paper has developed an evidence classification system to assess the reliability of each interaction. Based on the source of each interaction and the importance of its characteristic attributes, we have categorized all interactions into Levels 1 to 5, with Level 5 representing the interactions with the highest credibility. The following conditions are required to reach all levels:

Firstly, the MHC–peptide–TCR interaction was ranked according to the interaction information of MHC, peptide and TCR sequences that appeared in four databases VDJdb, TBAdb, McPAS-TCR and ATLAS. Entries that appeared in all four databases at the same time were assigned a score of 4, entries that appeared simultaneously in any three databases were assigned a score of 3, entries that appeared in any two databases at the same time were assigned a score of 2 and entries that were recorded in only one database were assigned a score of 1. The TCR is represented by the alpha and beta protein peptide chains of CDR3 on the TCR that bind most tightly to the antigen, as well as the genes TRAV, TRAJ, TRBV, TRBD, and TRBJ that encode the α and β chains of the TCR. Subsequently, for each entry, we combined other characteristic information of the target peptide chain to define its corresponding rank.

**Figure 2.**
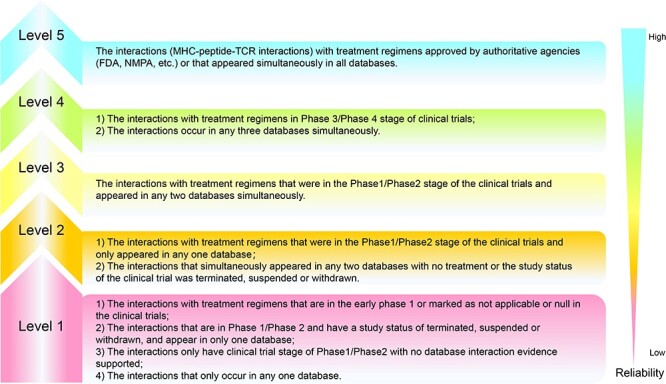
Reliability criteria for interactions in ACTs. Reliability is increasing from Levels 1 to 5, with each level having its own judgment criteria.

We define the interactions related to treatment regimens that have been specifically developed and approved by authoritative agencies (FDA, NMPA, etc.) or that appeared simultaneously in all databases as Level 5. For interactions related to treatment strategies in Phase 3/Phase 4 stage of the clinical trials or interactions that occur in any three databases simultaneously, we define them as Level 4. Level 3 was defined as an interaction if its treatment regimen was in the Phase 1/Phase 2 stage of the clinical trials and appeared in any two databases simultaneously. Level 2 was defined as the interaction that the treatment was in the Phase 1/Phase 2 stage of the clinical trials and only appeared in any one database or the interaction that simultaneously appeared in any two databases although there was no treatment or the study status of the clinical trial was terminated, suspended or withdrawn. For interactions related to treatment regimens that are in the early Phase 1 or marked as not applicable or null in ClinicalTrials.gov; or are in Phase 1/Phase 2 and have a study status of terminated, suspended or withdrawn, and appear in only one database; or only have clinical trial stage of Phase 1/Phase 2 with no database interaction evidence supported; or interactions that only occur in any one database are defined as Level 1. The specific code is implemented in Python. The rules for establishing the reliability criteria are shown in Figure 2.

### Database construction

The database DIRMC utilizes MySQL to store, query and manage all the included data. The web interface is constructed using JavaScript and HyperText Markup Language. Scripts for data processing programs are written in Python. Operations such as calling and fetching data from the database are achieved through Pymysql. The web service is hosted on Alibaba Cloud storage. DIRMC is tested in Mozilla Firefox, Google Chrome and Microsoft Edge browsers, which is freely accessible to the user community at website http://www.dirmc.tech/ and requires no registration or login.

## Results

### Database overview

**Figure 3.**
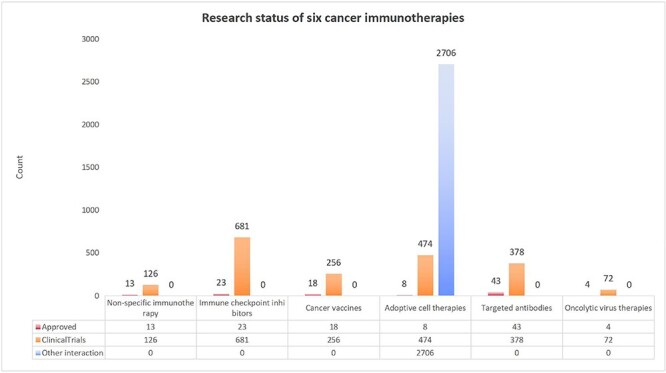
Data distribution for the six types of cancer immunity. In the figure, the horizontal axis represents six types of cancer immunotherapy and the vertical axis represents the quantity. The three bars for each therapy, from left to right, indicate approved therapies, therapies in clinical trials and the MHR–peptide–TCR interactions for ACTs, respectively.

As of March 2023, after integrating multiple different types of data resources and systematically reviewing 18 566 articles (Table 1), we had manually collected a total of 96 807 data entries. This includes 4802 entries related to cancer immunotherapy, 397 genes encoding target proteins, 4345 potentially pathogenic variants, 71 481 MHC–peptide–TCR interactions from experimental and high-throughput sequencing data resources, 7665 drug interactions with target genes from DGIdb database, 1865 target-related GO functions and 129 KEGG pathways, 8514 functional annotation information such as tissue types commonly enriched for each target protein, gene location, gene sequence, related molecular features from DrugBank and ChEMBL, including general function, specific function, etc., as well as sequence, regions and sites of amino acid etc. (Table 2). Among these entries, items related to cancer immunotherapy include those approved by authoritative agencies from various countries (such as FDA, NMPA, EDA, etc.), entries manually curated from ClinicalTrials.gov and literature and interaction relationships confirmed from experiments (Figure 3). You can access detailed data through the download interface of the database.

**Table 1.** Summary of statistical results based on data characteristics

Different types of information in the database	Sources with drug information	Count
Immunotherapy target interaction information	Targets collected in DGIdb	7665
	Approved by FDA/NMPA, etc.	109
	Clinical Trials/DrugBank	1987
	Entries that co-occur in three databases: VDJdb, McPAS-TCR, TBAdb/ATLAS, VDJdb, McPAS-TCR	3432
	Entries that co-occur in two databases: VDJdb, McPAS-TCR/VDJdb, TBAdb/VDJdb, ATLAS/McPAS-TCR, TBAdb	17 256
	Entries that appears in only one database: VDJdb/TBAdb/McPAS-TCR/ATLAS	50 793
Functional annotation of target proteins	GO/KEGG/ChEMBL/DrugBank/NCBI Gene/GenBank/UniProtKB	8514
Pathogenic variants in target genes	ClinVar	4345

**Table 2.** Statistical results of molecular characteristics contained in different data tables that can be downloaded

Data types	Number of features
Six cancer immunotherapies	4802
Pathogenic variants	4345
Target interactions from four data resources: VDJdb, McPAS-TCR, TBAdb and ATLAS	71 481
Target gene–drug interactions from DGIdb	7665
Functional annotation of target proteins	8514
Total	96 807

### User interface

DIRMC provides a user-friendly web interface for users, including ‘Search’, ‘Analysis’, ‘Overview’, ‘Download’, ‘Submit’ and ‘About’ sections.

The database ‘Home’ interface offers simple and fast exploration capabilities, including searching by keyword in the ‘Quick Search’ bar and browsing by different types of immunotherapy. Each type of immunotherapy can be directly accessed to their respective analysis interface, facilitating detailed characterization of the information involved in the target of each immunotherapy. To further assist users in expanding candidate MHC–peptide and peptide–TCR interactions for the study, we provide a schematic of the interaction between tumor cells and T cells in the ‘Potential interaction prediction’ section of the ‘Home’ page. In the schematic representation of the MHC–peptide–TCR interaction between two cell types, we provide two graphical interfaces that can predict MHC–peptide or peptide–TCR interaction, respectively. Users can predict the interaction for MHC–peptide or peptide–TCR by clicking on the graph of MHC–peptide or peptide–TCR. The ‘Analysis’ page also provides the corresponding quick selection interface.

On the left side of the ‘Search’ page, a list of molecular features associated with the target protein is provided, including disease name, cancer immunotherapy type, gene, target, the status of immunotherapy (approved or in clinical trial), and MHC or TCR information. Users can perform a quick search query for the information of interest by selecting a specific node in the directory tree, and all entries containing the search information are displayed in real time at the bottom of the page. Additionally, all data found on this page can be downloaded directly. When no specific node is selected, the ‘Result’ section at the bottom of the page is empty. Furthermore, DIRMC supports the ‘fuzzy’ search functionality, and the users can find all the results they are interested in by entering their keywords in the search box in the database. The corresponding results are also displayed in the data table at the bottom of the page.

**Figure 4.**
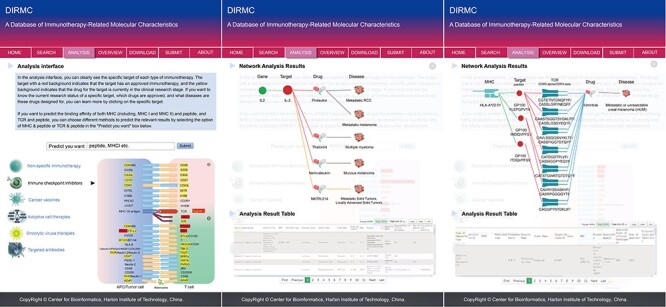
The analysis page of the database website. From left to right, the first figure shows the ‘Predict you want’ fast predictive interaction interface in the middle and the display of ICIs at the bottom. Figure two shows schematic diagram of the network analysis results and corresponding data table information when selecting the target protein for non-ACTs. Figure three shows the schematic diagram of network analysis results and corresponding data table information when selecting the target protein for TCR-T therapy of ACTs.

The ‘Analysis’ page allows users to gain insights into the molecular features related to cancer immunotherapy. By selecting the target of interest, users can visualize the associations between immunotherapy targets, genes, diseases and drugs. Detailed information of each term is displayed in the ‘Analysis Result Table’ at the bottom of the page. When clicking on molecular features such as genes and targets in the network, the contents in the following table will be updated synchronously with disease-related mutation information on the gene and the molecular functions, affected pathways, protein sequences, regions and sites affecting protein structure and other information related to the target protein. In the visualized network, the meaning of nodes from left to right differs for various types of therapies. For non-specific immunotherapy, ICIs, cancer vaccines, oncolytic virus therapies and targeted antibodies, the nodes from left to right in the visualized network diagram are genes, targets, drugs and diseases. For ACTs, there are two representations of the network. One is for TCR-T therapy, where the nodes from left to right in the visualized network diagram are MHC, target (peptide), TCR, drug and disease, as shown in Figure 4. For other types of therapies, such as CAR-T and chimeric antigen receptor natural killer cell therapy, the network diagram representation is similar to the aforementioned types of immunotherapies.

The ‘Overview’ page provides users with an overall flowchart of the database and a summary table of the data sources for all entries contained in the database, which facilitates users to gain a comprehensive understanding of the database. Additionally, it includes statistical histograms of the major molecular features associated with each type of immunotherapy. This can help users to have a systematic understanding of the fundamental attributes and quantitative relationships of molecular features related to cancer immunotherapy.

DIRMC allows users to download all cancer immunotherapy-related molecular feature data on the ‘Download’ page. In addition, DIRMC invites users to submit newly discovered molecular feature data related to cancer immunotherapy on the ‘Submit’ page to update the database. Once approved by the submission review committee, the submitted records will be included in the database and made available to the public in the subsequent release versions. If users encounter any issues while using the database, they can contact the email provided on the ‘About’ page for assistance.

## Discussion

With continuous breakthroughs in medical advancements for cancer treatment and ongoing deepening of researchers’ understanding of cancer, cancer immunotherapy has been developing vigorously. From 1972 to the present, it has exhibited an explosive growth trend. In the past decades, data related to cancer immunotherapy have accumulated rapidly. In 2013, the journal ‘Science’ ranked cancer immunotherapy as the first of the top 10 scientific breakthroughs (30). In the past decade, immunotherapy has been hailed as the most promising approach to conquer cancer (31). A comprehensive understanding of the molecular characteristics related to cancer immunotherapy is crucial to further explore the potential molecular interaction mechanism, develop new algorithms to predict the molecular interaction, stay updated on the cutting-edge directions in cancer immunotherapy and develop novel cancer immunotherapy. Most of the existing immunological databases focus on algorithms for predicting binding affinity or only concentrate on molecular interaction of MHC–peptide–TCR. There is still a lack of public databases centered around molecular features related to immunotherapy.

To meet this need, we have developed DIRMC, a database of immunotherapy-related molecular characteristics. It comprehensively characterizes all molecular features involved in different types of cancer immunotherapy, from the original gene sequence information, to the pathogenic variant characteristics, genes, peptide sequences, protein sequences, and the corresponding disease types, the status of corresponding therapies, including whether they are approved for use or in clinical trials, the manufacturer, and the study status and phase of the clinical trials, etc. In addition, DIRMC provides a prediction interface that allows users to predict the interaction of peptides of interest with MHC or TCR. Users can choose different methods, such as the 1D Convolutional Neural Networks-based model or a comprehensive k-mer matching approach, etc.; by submitting the amino acid sequence(s) of the peptide(s) of interest, the binding affinity of peptide to MHC I or MHC II or TCR can be obtained.

Therefore, DIRMC is a user-friendly dedicated database that provides a comprehensive resource on molecular features related to cancer immunotherapy. It will be of particular interest to both the general researchers and the broader life sciences community. In future updates, we will continue the approach of integrating manually curated and validated molecular interactions related to cancer immunotherapy and reviewing the submission on the web page. The database will be updated every 2 months to continually expand its content. In addition, our in-house MHC–peptide binding affinity prediction algorithm for ACT therapy will be added to the analysis page as a user-friendly tool. We will also provide interfaces for other prediction tools to expand the analysis and prediction capabilities of our database, enabling users to upload their own data to get a predictive result. Finally, we will continue to optimize our database to provide users with an enhanced user experience.

We believe that as more data and predictive tools are integrated into DIRMC, it will contribute to further research into the molecular interaction mechanisms of cancer immunotherapy, exploring potential molecular interactions, developing new algorithms for predicting molecular interactions and advancing the development of new cancer immunotherapy.

## Data Availability

This database can be freely accessed via website http://www.dirmc.tech/.

## Footnotes

1. Zhang W, Wang L, Liu K, Wei X, Yang K, Du W, Wang S, Guo N, Ma C, Luo L, Wu J, Lin L, Yang F, Gao F, Wang X, Li T, Zhang R, Saksena NK, Yang H, Wang J, Fang L, Hou Y, Xu X, Liu X. PIRD: Pan Immune Repertoire Database. Bioinformatics. 2020 Feb 1;36(3):897–903. doi: 10.1093/bioinformatics/btz614. PMID: 31373607.
